# Atypical vocal imitation of speech and song in autism spectrum disorder: Evidence from Mandarin speakers

**DOI:** 10.1177/13623613241275395

**Published:** 2024-09-06

**Authors:** Li Wang, Peter Q Pfordresher, Cunmei Jiang, Fang Liu

**Affiliations:** 1 The Chinese University of Hong Kong, China; 2 University of Reading, UK; 3 University at Buffalo, The State University of New York, USA; 4 Shanghai Normal University, China

**Keywords:** acoustics, autism, song, speech, vocal imitation

## Abstract

Vocal imitation in English-speaking autistic individuals has been shown to be atypical. Speaking a tone language such as Mandarin facilitates vocal imitation skills among non-autistic individuals, yet no studies have examined whether this effect holds for autistic individuals. To address this question, we compared vocal imitation of speech and song between 33 autistic Mandarin speakers and 30 age-matched non-autistic peers. Participants were recorded while imitating 40 speech and song stimuli with varying pitch and duration patterns. Acoustic analyses showed that autistic participants imitated relative pitch (but not absolute pitch) less accurately than non-autistic participants for speech, whereas for song the two groups performed comparably on both absolute and relative pitch matching. Regarding duration matching, autistic participants imitated relative duration (inter-onset interval between consecutive notes/syllables) less accurately than non-autistic individuals for both speech and song, while their lower performance on absolute duration matching of the notes/syllables was presented only in the song condition. These findings indicate that experience with tone languages does not mitigate the challenges autistic individuals face in imitating speech and song, highlighting the importance of considering the domains and features of investigation and individual differences in cognitive abilities and language backgrounds when examining imitation in autism.

**Lay abstract:**

Atypical vocal imitation has been identified in English-speaking autistic individuals, whereas the characteristics of vocal imitation in tone-language-speaking autistic individuals remain unexplored. By comparing speech and song imitation, the present study reveals a unique pattern of atypical vocal imitation across speech and music domains among Mandarin-speaking autistic individuals. The findings suggest that tone language experience does not compensate for difficulties in vocal imitation in autistic individuals and extends our understanding of vocal imitation in autism across different languages.

## Introduction

Imitation is an essential aspect of skill development (Hurley & Chater, 2005). In the first few years of life, children rapidly learn new skills, such as the typical uses of certain things and the basics of their mother tongue. The rapid learning abilities of young children can be attributed, in part, to humans’ remarkable capacity to imitate what they see and hear (Tomasello et al., 1993). Starting from infancy, typically developing children learn to imitate others’ object-directed actions, gestures, body movements, and sounds or words (Meltzoff, 2017). The process of imitating others or being imitated not only facilitates the development of skills but also lays the foundation for interaction and communication with others, for example, by expressing interests in their caregivers or peers, sharing emotions as well as paying attention to others (Ingersoll, 2008; Uzgiris, 1981).

However, deviations in imitation, especially in the vocal domain, can exert a profound impact on the development of social interaction and communication, as exemplified in autism spectrum disorder (ASD; Chen et al., 2022; Diehl & Paul, 2012; Fosnot & Jun, 1999; Hubbard & Trauner, 2007; Paul et al., 2008; Van Santen et al., 2010; Wang, Pfordresher, et al., 2021). Research has shown that autistic and non-autistic individuals differ in how they vocally imitate sounds and speech, particularly in terms of pitch and duration patterns. For example, when autistic individuals try to imitate prosodic patterns, such as making a sentence sound like a question or a statement, or expressing likes or dislikes, they often exhibit prolonged durations of the sentences compared to their non-autistic peers (Diehl & Paul, 2012; Paul et al., 2008). In addition, autistic individuals tend to use a higher pitch when imitating the stress patterns in nonsense words (i.e. make-up words, like “*tauveeb*”) than non-autistic individuals (Van Santen et al., 2010). Studies also find that when autistic individuals imitate speech to convey statements, questions, or emotions, their patterns are different from those of non-autistic individuals in both pitch and duration characteristics (Fosnot & Jun, 1999; Hubbard & Trauner, 2007; Wang, Pfordresher, et al., 2021). Understanding these acoustic differences (e.g. pitch and duration) in vocal imitation can inform the development of more effective communication strategies and interventions for autistic individuals (Mazaheri & Soleymani, 2018).

Notably, the majority of these investigations have been conducted with speakers of non-tonal languages, and the literature lacks representation from speakers of tone languages. The world’s languages can be classified into tone (e.g. Mandarin, Cantonese) versus non-tonal (e.g. English) languages, depending on how they use pitch to convey meaning (Xu, 2019; Yip, 2002). Specifically, across tone and non-tonal languages, pitch is used to convey prosodic meaning (Krishnan & Gandour, 2009), including intonation such as statement-question intonation (Wang, Beaman, et al., 2021) and emotions like excitement and sadness (Rodero, 2011). However, pitch additionally serves a lexical function of distinguishing different word meanings in tone languages (Klein et al., 2001). For example, with the same syllable /ma/, the word 妈 with a high-level tone (i.e. Tone 1 in Mandarin) means “mother,” whereas the word 马 with a falling-rising tone (i.e. Tone 3 in Mandarin) means “horse.” Thus, unlike in English, the imitation of pitch-related features in Mandarin occurs in parallel, with prosodic meaning represented at the sentence level and lexical meaning at the syllable or word level (F. Liu & Xu, 2005; Yuan, 2011). Due to the additional role pitch plays in tone languages, enhanced pitch processing abilities in tone language speakers have been widely demonstrated (see J. Liu et al. (2023) for review), underscoring the need to explore vocal imitation in autistic individuals within tonal linguistic contexts.

**Figure 1.**
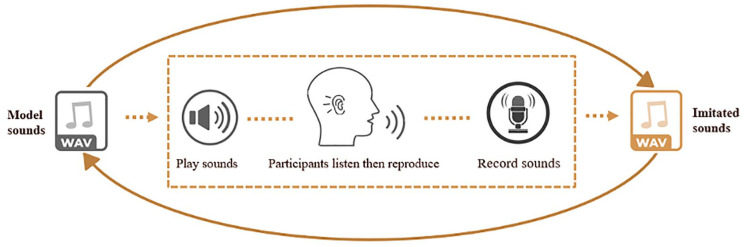
The illustration of vocal imitation process.

This study, therefore, seeks to provide a more nuanced exploration of vocal imitation, specifically among autistic Mandarin speakers. In addition to addressing the lacunae in existing literature, we also examined the matching between the model and imitated sounds, a critical measure of imitation accuracy that is often overlooked in previous acoustic studies. As depicted in Figure 1, without considering model sounds, a direct comparison of the acoustic features (e.g. pitch and duration) of the imitated sounds between the autistic and non-autistic groups provided insights solely into the characteristics of imitated sounds, rather than imitation accuracy. This oversight failed to capture participants’ vocal imitation ability *per se*, that is, the ability to match the acoustic features of the model sounds through imitation (Mercado et al., 2014; Wang, Pfordresher, et al., 2021). Comparing imitated sounds to the original targets offers valuable insights into the nature of vocal imitation differences in autism. This, in turn, can inform targeted clinical interventions and contribute to the broader understanding of vocal imitation abilities in autism. In an effort to fill this gap, our previous study examined speech and song imitation in an English-speaking sample, who were instructed to imitate exactly the pitch and timing patterns of the sentences they heard (i.e. Model sounds) while their voices were being recorded (i.e. Imitated sounds) (Wang, Pfordresher, et al., 2021). The vocal imitation ability was measured by comparing the pitch- and duration-related parameters between the model and the imitated sounds, with smaller differences indicating more accurate imitation. Results revealed that vocal imitation differences exist among English-speaking autistic individuals across speech and music domains, especially in terms of absolute pitch and duration matching (Wang, Pfordresher, et al., 2021).

Using the same paradigm, the current study strived to deepen the insights into vocal imitation among Mandarin-speaking autistic individuals. Through acoustic analysis, we aimed to quantify speech and song imitation abilities of Mandarin-speaking autistic and non-autistic individuals, addressing the following questions: (1) Do imitation abilities of Mandarin-speaking autistic individuals differ from non-autistic individuals in terms of pitch-related features across speech and music domains? (2) Do Mandarin-speaking autistic individuals differ from non-autistic individuals with respect to duration-related feature matching in vocal imitation? Based on the differences in how pitch is used in Mandarin and English speech, we hypothesized that vocal imitation of pitch-related features in Mandarin-speaking autistic individuals may not be affected, unlike English speakers. This expectation arose from the elevated sensitivity and proficiency in processing pitch observed in Mandarin speakers (J. Liu et al., 2023). Regarding duration-related features, a cross-linguistic study found that machine learning using speech rhythm can differentiate autistic from non-autistic individuals across English and Cantonese, suggesting that speech rhythm is an important feature of autism that is evident in multiple languages (Lau et al., 2022). We therefore predicted that, like English speakers, Mandarin-speaking autistic individuals may have difficulty in imitating duration patterns in both speech and music. Based on previous findings on English speakers (Wang, Pfordresher, et al., 2021), we also hypothesized that Mandarin-speaking autistic participants would show poorer performance on absolute feature matching, but not relative feature matching as compared to non-autistic participants.

## Method

### Participants

A group of 33 autistic children (aged between 7 and 16) and 30 age-matched non-autistic children took part in the study. All were native speakers of Mandarin and reported no history of other neurological or psychiatric disorders. They were recruited from special educational facilities and mainstream schools in Nanchang and Nanjing, China. The autistic children all had a clinical diagnosis of autism using either *DSM*-IV or *DSM*-5 (American Psychiatric Association, 1994, 2013) which was further supported by the Autism Diagnostic Observation Schedule—Second Edition (ADOS-2) (Lord et al., 2012) conducted by the first author (with research reliability for administration and scoring). All autistic participants were administered the ADOS-2 Module 3 according to their developmental and language levels. Total scores on the ADOS-2 were converted to a comparative score (CS) of 1–10, with 10 representing the highest severity of autism-related symptoms (Duda et al., 2014; Gotham et al., 2009). All participants had normal hearing in both ears, with pure-tone air conduction thresholds of 25 dB HL or better at frequencies of 0.5, 1, 2, and 4 kHz, as assessed using an Amplivox manual audiometer (Model 116). Participants completed a nonverbal IQ test using the Raven’s Standard Progressive Matrices Test (RSPM) (Raven et al., 1998) and a receptive vocabulary test using the Chinese version of the Peabody Picture Vocabulary Test-Revised (PPVT-R) (Dunn & Dunn, 1981; Sang & Miao, 1990). The standardized scores for RSPM and PPVT-R were calculated as described by Wang et al. (2023). For RSPM, the standardized scores were derived using the means and standard deviations from a Chinese normative study (Zhang, 1989). As the Chinese norms for PPVT-R covered only ages 3.5 to 9 (Sang & Miao, 1990), we used American norms (Dunn & Dunn, 1981) to calculate the standardized scores. A correlation analysis showed a significant positive relationship (*r* = 0.95) between the standardized scores based on the Chinese norms and those based on the American norms for participants aged 9 and below, validating this methodology. The Chinese version of the forward digit span task was used to assess verbal short-term memory (Wechsler, 2003). Participants’ musical training background and their ability to identify a musical note without a reference tone (i.e. absolute pitch or perfect pitch) (Deutsch, 2013) were collected using a caregiver-reported questionnaire, and their years of formal musical training were summed across all instruments including voice (Wang, Beaman, et al., 2021). Participants’ perceptual skills were assessed using a statement-question intonation discrimination task, taken from a comparative study investigating speech and music perception (F. Liu et al., 2012; Wang et al., 2023). As can be seen in Table 1, the results of Welch’s *t*-test showed that the autistic and non-autistic groups were comparable on all background measures, except the PPVT-R scores, which were taken into account in the statistical models.

**Table 1.** Characteristics of the autism (*n* = 33) and non-autism groups (*n* = 30).

Background measures	Autism	Non-Autism	*t*	*p*	Cohen’s *d*
Gender (F:M)	5:28	4:26			
Age	10.29 (2.50)	11.50 (2.83)	1.79	0.08	0.45
Musical training	0.88 (1.32)	0.50 (1.11)	1.24	0.22	0.31
RSPM	110.12 (15.77)	112.72 (10.26)	0.78	0.44	0.20
PPVT-R	**124.33 (25.87)**	**141.77 (12.80)**	**3.44**	0.001**	**0.85**
Digit span	8.49 (0.91)	8.07 (1.11)	1.63	0.11	0.41
Self-reported absolute pitch	*n* = 2	*n* = 3			
Perception-Natural speech	1.57 (0.87)	1.81 (0.75)	1.19	0.24	0.30
Perception-Gliding tone	1.60 (0.86)	1.93 (0.63)	1.71	0.09	0.43
ADOS-CS	6.97 (2.31)	NA			

*Note*. Musical training: years of musical training; RSPM: standard score of Raven’s Standard Progressive Matrices Test; PPVT-R: standard score of Peabody Picture Vocabulary Test-Revised; Digit span: raw score of verbal short-term memory; Perception-Natural speech and Perception-Gliding tone: D-prime values for subtest scores, with higher values representing better perception skill; ADOS-CS: comparative score of ADOS, with 10 representing the highest severity of autism-related symptoms. Bold values indicate statistical significance at *p* < 0.05. *p < 0.05, **p < 0.01, ***p < 0.001.

### Community involvement

There was no community involvement in the present study.

### Stimuli

**Figure 2.**
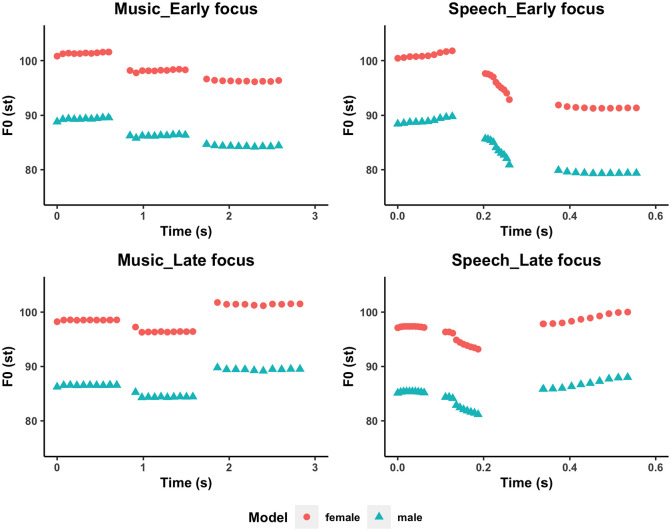
The pitch-time trajectory of the sentence “她的包? vs. 她的包? (Ta1 de0 bao1?/ Her bag?) under different conditions by female/male model speakers.

The model stimuli were 10 sentences either spoken or sung with an early focus or a late focus from F. Liu et al. (2013), yielding 40 sentences with two to six syllables each (see Table 2 for the list of sentences and Supplementary Table 1 for musical notations of the sung stimuli). The inclusion of different sentence lengths was to control for the effect of stimulus length on imitation performance (F. Liu et al., 2013). The manipulation of the different focus conditions of the sentences ensured the inclusion of a variety of pitch and duration patterns in the speech stimuli, as focused words normally show a higher pitch and longer duration than their unfocused counterparts in Mandarin speech (F. Liu & Xu, 2005; Yuan, 2011). For example, in the top right panel of Figure 2, the sentence “**她**的包?” [“**Her** bag?”] has an initial focus on the word “**她**” [“**Her**”] which has a higher pitch and longer duration than the same unfocused word in the bottom right panel of Figure 2, where the sentence “她的**包**?” [“‘Her **bag**?”] has a final focus on the word “**包**” [“**bag**”]. As can be seen from the top and bottom left panels of Figure 2, the corresponding song stimuli approximated the global melodic contours and timing variations of the speech stimuli. To both accommodate participants’ vocal range and to ensure that participants of different ages or gender were exposed to the same pitch and duration patterns of the speech/song stimuli, we adopted the male and female versions of the stimuli from F. Liu et al. (2013). The female model was originally recorded by a 27-year-old Mandarin-speaking female student who was born and raised in Beijing. To ensure that the stimuli encountered by male and female participants have identical pitch intervals and rhythmic patterns, the female model was synthesized (preserving the absolute pitches and formant frequencies of the original recordings) and the male model was generated from the female model by changing the original pitches to one octave lower and shifting the frequencies of the original formants by .78 to achieve male voice characteristics, using the “change gender” command in Praat (Boersma & Weenink, 2001). The ecological validity of the synthesized female and male models was tested and confirmed in F. Liu et al. (2013), where Mandarin-speaking female and male adult participants with and without congenital amusia performed the same imitation task using the same stimulus set. None of the participants in F. Liu et al. (2013) noted any unnaturalness of the stimuli, and no significant differences were found in imitation performance between the participants of different genders for either the amusic or the non-amusic group. Thus, the current study adopted the same stimulus set as in F. Liu et al. (2013). We also did not observe any significant differences in imitation performance across female and male participants in the current sample (see Supplementary Table 2). The male version was used for male participants ⩾12 years old, and the female version was used for female participants regardless of age as well as male participants < 12 years old, as research indicates that children up to 12 show similar pitch ranges (Mecke & Sundberg, 2010; Nicollas et al., 2008; Sergeant & Welch, 2009).

**Table 2.** Stimuli used in the experiment.

Stimuli with an early focus	Stimuli with a late focus	Chinese Pinyin	English translation
**黑**车？	黑**车**？	Hei1 che1?	Black car?
**青**天？	青**天**？	Qing1 tian1?	Blue sky?
**她**的包？	她的**包**？	Ta1 de0 bao1?	Her bag?
**三**颗星？	三**颗**星？	San1 ke1 xing1?	Three stars?
**冬天**的风？	冬天的**风**？	Dong1 tian1 de0 feng1?	The winter’s wind?
写**他**书上？	写他**书**上？	Xie3 ta1 shu1 shang0?	Write on his book?
**漆黑**的天空？	漆黑的**天空**？	Qi1 hei1 de0 tian1 kong1?	Pitch-black sky?
小**丁**长高了？	小丁长**高**了？	Xiao3 ding1 zhang3 gao1 le0	Xiao Ding grew taller?
老**郭**的猫丢了？	老郭的**猫**丢了？	Lao3 guo1 de0 mao1 diu1 le0	Lao Guo’s cat is lost?
小方**天天**加班？	小方天天**加班**？	Xiao3 Fang1 tian1 tian1 jia1 ban1?	Xiao Fang works overtime every day?

### Procedure

The presentation of the model stimuli and the recording of the imitations were both done using Praat (Boersma & Weenink, 2001). Participants were seated in a quiet room and were presented with four practice trials (with items different from those in experimental trials: 2 speech vs 2 song) to familiarize themselves with the task and the recording environment. Following the practice section, participants were presented with each of the 40 speech/song sentences one at a time in pseudorandom order and were instructed to imitate exactly the pitch and timing patterns of the sentences to the best of their ability, while their voices were recorded via a Roland RUBIX22 USB Audio Interface. Each sentence was played once and only replayed when participants failed to catch the words, and not when they wanted to listen to it again so they could imitate it better.

### Data analysis

Recordings were analyzed in Praat using ProsodyPro, a software tool designed for the automatic analysis of extensive speech data (Xu, 2013). To ensure precise acoustic measurements, we adopted a hybrid approach. This involved initial automated processes using ProsodyPro and subsequent manual verification by trained phoneticians (authors LW and FL) to extract the pitch and duration of each syllable rhyme. Syllable/note duration was calculated as the length of the syllable rhyme, and the onset of syllable rhyme was defined as the syllable/note onset time. The median F0s (fundamental frequencies) of the syllable rhymes were extracted to indicate pitch heights. Octave errors in pitch imitation were corrected, that is, when the imitated pitch was more than 6 semitones (half octave) apart from the model pitch, the value was adjusted as 12—imitated pitch. In total, less than 4.11% of the data samples needed to be adjusted, and most of these errors were caused by creaky voices, resulting in decreased F0 (Johnson, 2011). Trained phoneticians manually added these missed vocal pulse marks for F0 based on the waveforms and spectrograms, to avoid having erroneous outliers misleading imitation results.

We used absolute pitch and duration matching to refer to the ability to imitate individual syllables/notes based on their acoustic features, irrespective of their relationship with surrounding syllables/notes. In addition, following F. Liu et al. (2013) and previous singing or pitch-matching studies (Dalla Bella et al., 2007, 2009, 2011; Pfordresher & Brown, 2007; Pfordresher et al., 2010; Ward & Burns, 1978), we also measured the number of pitch contour, pitch interval, and time errors that deviated from the corresponding model’s pitch direction or specific pitch interval or duration value. The pitch was measured in “cents” (100 cents = one semitone), a unit of measure based on the equal-tempered scale in music, to facilitate a more nuanced representation of pitch distinctions and a finer resolution in the assessment of pitch differences. Detailed definitions of these measures are provided below.

**The absolute pitch deviation (in cents):** Median F0 was extracted from each syllable rhyme and then subtracted from that of their matched model to find the pitch deviation (in absolute value) for each imitated rhyme. The deviations were averaged over all syllables/notes in each utterance/melody and the bigger the value, the less accurate the imitation in terms of absolute pitch matching.

**The relative pitch deviation (in cents):** The pitch interval was calculated as the absolute difference in median F0 between two consecutive syllables/notes, and then subtracted from their matched model’s pitch interval (in absolute value). The deviations were averaged over all intervals in each utterance/melody and the bigger the value, the less accurate the imitation in terms of relative pitch matching.

**The number of pitch contour errors:** Pitch contour errors were defined as imitated pitch intervals that differed from the corresponding model pitch intervals regarding pitch directions (up, down, or level). Pitch direction was considered to be up or down if the difference in pitch interval was higher or lower by 50 cents or more; otherwise (the difference was within 50 cents), the pitch intervals were considered to form a level/flat pitch direction. The number of contour errors was summed over each utterance/melody.

**The number of pitch interval errors:** Pitch interval errors were defined as imitated pitch intervals that were larger or smaller than the corresponding model pitch intervals by 100 cents without considering the pitch direction. Specifically, imitated and model pitch intervals were compared using absolute values. The number of pitch interval errors was summed over each utterance/melody.

**The absolute duration difference (in milliseconds):** Duration was extracted from each syllable rhyme and then subtracted from their matched model’s production to find the absolute difference for each rhyme. The differences were averaged over all rhymes in each utterance/melody and the larger the value, the less accurate the imitation in terms of absolute duration matching.

**The relative duration difference (in milliseconds):** Interonset interval (IOI) was calculated as the difference between the onsets of two consecutive syllables/notes, and then subtracted from their matched model’s IOI (in absolute value). The differences were averaged over all IOIs in each utterance/melody and the larger the value, the less accurate the imitation in terms of relative duration matching.

**The number of time errors:** Time errors were defined as imitated syllables/notes that were more than 25% longer or shorter than the corresponding model syllables/notes (Dalla Bella et al., 2007, 2009; Prince & Pfordresher, 2012). In Western tonal music, the durations of different events such as sixteenth notes (1/4 a beat), eighth notes (1/2 a beat), and quarter notes (1 beat) are in simple integer ratio relationships (Drake & Palmer, 2000). Similarly, speech rhythm can also be measured in relative terms (Patel & Daniele, 2003; Patel et al., 2006). Thus, using a 25% deviation to count time errors not only captures the violation of the time signature in music but also makes the comparison of spoken and musical rhythm possible. The number of time errors was summed over each utterance/melody.

All statistical analyses were conducted using Rstudio (RStudio Team, 2020). We performed linear mixed-effects analysis, which is robust to violations of statistical assumptions (Gelman & Hill, 2006; Schielzeth et al., 2020). The *lme4* (Bates et al., 2012; Brauer & Curtin, 2018) and *lmerTest* (Kuznetsova et al., 2017) packages were used with the above-mentioned pitch and duration variables as the dependent variable and Group (effect-coded: Non-autism vs Autism), and Condition (effect-coded: Speech vs Music) as well as the interaction between Group and Condition as fixed effects. To take into account the significant group differences in receptive vocabulary and the relatively wide age range, we also added PPVT-R scores and age (both variables were mean-centered) in the models. Years of musical training were significantly associated with only one of the pitch metrics: More musical training was associated with fewer pitch interval errors (*B* = -0.06, SE*B* = 0.03, *t*(61.41) = -2.26, *p* = 0.03). Thus, in the interest of space, musical experience was not considered in the models. All models were fit using the maximal random effects structure that converged with two random factors (subject vs item) (Barr, 2013; Barr et al., 2013). When the maximal model failed to converge, the random correlations were removed first. If the model still failed to converge, the random effect with the lowest variance was iteratively removed until the model converged. Subsequent post hoc comparisons, if any, were conducted using the *emmeans* package with Holm-Bonferroni correction for multiple comparisons (Lenth et al., 2018).

## Results

### Absolute pitch deviation

**Figure 3.**
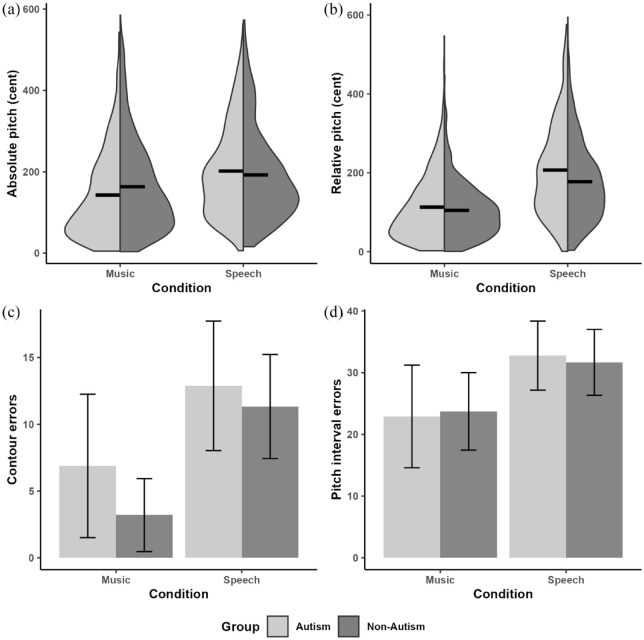
Pitch-related measures for the autism and non-autism groups. (a) Absolute pitch deviations (in cents), with black lines representing mean values. (b) Relative pitch deviations (in cents), with black lines representing mean values. (c) The number of pitch contour errors, with error bars representing the standard deviation. (d) Number of pitch interval errors, with error bars representing the standard deviation. Different plots are selected depending on the nature of the data type, with (a) and (b) representing continuous data, (c) and (d) representing discrete data.

Figure 3(a) shows the distribution of absolute pitch deviations for each group in both the Speech and the Music conditions. These values were obtained by averaging the absolute pitch deviations across the syllables/notes (ranging from two to six) within each of the 20 utterances/melodies produced by each participant. These averages captured participants’ performance across the entire stimuli while minimizing the variations caused by extreme values (e.g. due to creaky voice). Results revealed a main effect of Condition (*B* = −22.55, SE*B* = 5.49, *t*(31.54) = −4.11, *p* < 0.001) and a Group * Condition interaction (*B* = −7.78, SE*B* = 3.36, *t*(51.99) = −2.31, *p* = 0.02). Post hoc analyses with Holm-Bonferroni correction for multiple comparisons suggested no group differences in either condition (Speech: *t*(72.8) = 0.17, *p* = 0.88; Music: *t*(72.6) = 1.67, *p* = 0.10); instead, the interaction was driven by both groups performing better on absolute pitch matching for music than for speech, with the trend being more pronounced in the autism group (*t*(48.5) = 4.76, *p* < 0.001, Music: M(*SD*) = 142.8(108.29); Speech: M(*SD*) = 201.96(110.98)) than in the non-autism group (*t*(50.9) = 2.27, *p* = 0.03, Music: M(*SD*) = 163.31(113.68); Speech: M(*SD*) = 192.22(112.98)). No other remaining main effects were significant (Table 3).

**Table 3.** Coefficients for the linear mixed-effects models for pitch-related measures.

Measure	Effect	Estimate	Std. Error	df	*t*	*p*
Absolute pitch deviation	Group	–9.47	9.76	59.03	–0.97	0.34
	Condition	–**22.55**	**5.49**	**31.54**	–**4.11**	**<** 0.001***
	PPVT-R	–0.64	0.43	59.01	–1.48	0.14
	Age	–3.44	3.34	59.04	–1.03	0.31
	Group × Condition	–**7.78**	**3.36**	**51.99**	–**2.31**	0.02*
Relative pitch deviation	Group	7.04	5.25	58.70	1.34	0.19
	Condition	–**44.77**	**9.02**	**23.89**	–**4.97**	**<** 0.001***
	PPVT-R	–0.36	0.23	58.64	–1.55	0.13
	Age	–2.89	1.79	58.76	–1.61	0.11
	Group × Condition	–**7.20**	**3.41**	**60.62**	–**2.11**	0.04*
Pitch contour errors	Group	**0.06**	**0.02**	**57.72**	**2.63**	0.01*
	Condition	**–0.18**	**0.04**	**24.82**	**–4.13**	**<** 0.001***
	PPVT-R	–0.0006	0.001	57.64	–0.60	0.55
	Age	–0.009	0.008	57.86	–1.09	0.28
	Group × Condition	0.02	0.02	60.66	1.05	0.30
Pitch interval errors	Group	–0.01	0.04	55.82	–0.30	0.76
	Condition	**–0.24**	**0.06**	**23.50**	**–4.16**	**<** 0.001***
	PPVT-R	–0.002	0.002	58.91	–1.06	0.29
	Age	**–0.03**	**0.01**	**59.10**	**–2.60**	0.01*
	Group × Condition	–0.04	0.02	60.91	–1.68	0.10

Bold values indicate statistical significance at *p* < 0.05. *p < 0.05, **p < 0.01, ***p < 0.001.

### Relative pitch deviation

Figure 3(b) shows the distribution of the relative pitch deviations for each group in both the Speech and the Music conditions. Results revealed a significant main effect of Condition (*B* = −44.77, SE*B* = 9.02, *t*(23.89) = −4.97, *p* < 0.001) and a significant interaction between Group and Condition (*B* = −7.20, SE*B* = 3.41, *t*(60.62) = −2.11, *p* = 0.04). Post hoc analyses with Holm-Bonferroni correction for multiple comparisons suggested that both groups showed better relative pitch matching for music than for speech (Autism: *t*(29.9) = 5.42, *p* < 0.001; Non-autism: *t*(31.1) = 3.88, *p* < 0.001), and the autism group performed worse than the non-autism group in the speech condition (*t*(102) = −2.27, *p* = 0.03, Autism: M(*SD*) = 215.08(133.52); Non-autism: M(*SD*) = 179.73(107.35)) but not in the music condition (*t*(102) = 0.03, *p* = 0.98, Autism: M(*SD*) = 113.88(86.71); Non-autism: M(*SD*) = 104.87(68.31)). No other remaining main effects were significant (see Table 3).

### Number of pitch contour errors

Figure 3(c) shows the distribution of the number of pitch contour errors for each group in both the Speech and Music conditions. These values were obtained by summing errors over two to six syllables/notes within each of the 20 utterances/melodies produced by each participant. Results revealed, as shown in Table 3, significant main effects of Group (*B* = 0.06, SE*B* = 0.02, *t*(57.72) = 2.63, *p* = 0.01) and Condition (*B* = −0.18, SE*B* = 0.04, *t*(24.82) = -4.13, *p* < 0.001), as both groups made fewer contour errors with the music condition (Autism: M(*SD*) = 6.88(5.37), Non-autism: M(*SD*) = 3.20(2.73)) than the speech condition (Autism: M(*SD*) = 12.88(4.85), Non-autism: M(*SD*) = 11.33(3.90)), and the autism group exhibited more pitch contour errors than the non-autism group across both conditions. The interaction between Group * Condition and the effects of PPVT-R and Age were not significant.

### Number of pitch interval errors

Figure 3(d) shows the distribution of the number of pitch interval errors for each group in both the Speech and Music conditions. As shown in Table 3, the linear mixed-effects model revealed a significant main effect of Condition (*B* = -0.24, SE*B* = 0.06, *t*(23.50) = -4.16, *p* < 0.001), as both groups showed fewer pitch interval errors in the music condition (M(*SD*) = 23.3(7.37)) than in the speech condition (M(*SD*) = 32.24(5.46)). Age was a significant predictor of the performance on pitch interval errors (*B* = −0.03, SE*B* = 0.01, *t*(59.10) = -2.60, *p* = 0.01), with older age associated with fewer interval errors. No other remaining main effects or interactions were significant. In addition, Pearson correlations confirmed the significant association between pitch interval errors and age (*r*(124) = −0.21, *p* = 0.02), but not with PPVT-*R* (*r*(124) = −0.04, *p* = 0.59).

### Absolute duration difference

**Figure 4.**
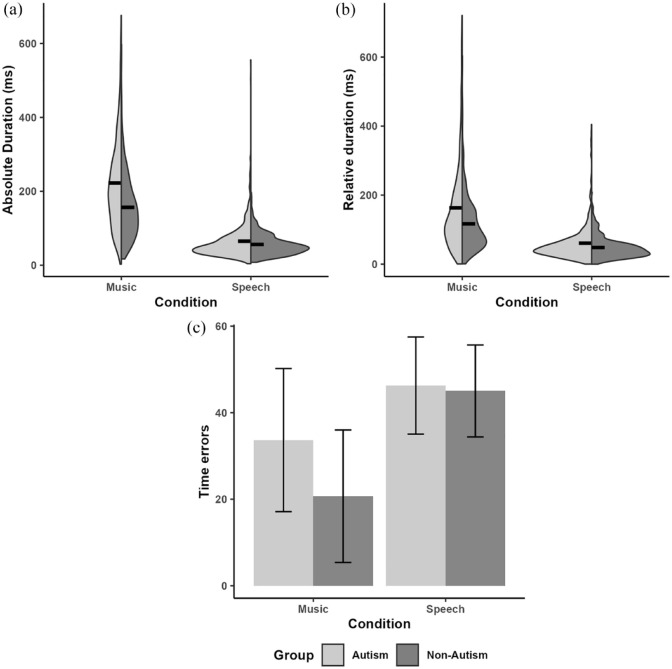
Duration-related measures for the autism and non-autism groups. (a) Absolute duration differences (in milliseconds), with black lines representing mean values. (b) Relative duration differences (in milliseconds), with black lines representing mean values. (c) Number of time errors, with error bars representing the standard deviation. Different plots are selected depending on the nature of the data type, with (a) and (b) representing continuous data, and (c) representing discrete data.

Figure 4(a) shows the distribution of the absolute duration differences for each group in both the Speech and Music conditions. The linear mixed-effects model revealed, as shown in Table 4, significant main effects of Group (*B* = 13.95, SE*B* = 4.91, *t*(58.81) = 2.84, *p* = 0.006), Condition (*B* = 64.65, SE*B* = 4.61, *t*(61.02) = 14.03, *p* < 0.001), as well as a Group * Condition interaction (*B* = 14.35, SE*B* = 4.61, *t*(61.02) = 3.11, *p* = 0.003). Post hoc analyses with Holm-Bonferroni correction for multiple comparisons suggested that both groups showed larger absolute duration differences in the music condition than in the Speech condition (Autism: *t*(61.1) = −12.41, *p* < 0.001; Non-Autism: *t*(60.9) = −7.54, *p* < 0.001, and the autism group produced larger absolute duration differences than did the non-autism group in the music condition (*t*(119) = −4.21, *p* < 0.001, Autism: M(*SD*) = 222.42(121.64); Non-Autism: M(*SD*) = 156.5(84.82)) but not in the speech condition (*t*(119) = 0.06, *p* = 0.95, Autism: M(*SD*) = 64.99(50.05); Non-Autism: M(*SD*) = 56.08(29.31)). Receptive vocabulary was a significant predictor of the performance on absolute duration matching (*B* = −0.8, SE*B* = 0.22, *t*(58.76) = −2.71, *p* = 0.009), with larger vocabulary associated with greater accuracy in absolute duration matching. The effect of Age was not significant. Again, Pearson correlations confirmed the significant association between the absolute duration differences and PPVT-*R* (*r*(124) = −0.22, *p* = 0.02), but not with age (*r*(124) = −0.02, *p* = 0.79).

**Table 4.** Coefficients for the linear mixed-effects models for duration-related measures.

Measure	Effect	Estimate	Std. Error	df	*t*	*p*
Absolute duration difference	Group	**13.95**	**4.91**	**58.81**	**2.84**	0.006**
	Condition	**64.65**	**4.61**	**61.02**	**14.03**	**<** 0.001***
	PPVT-R	**–0.58**	**0.22**	**58.76**	**–2.71**	0.009**
	Age	0.19	1.68	58.85	0.11	0.91
	Group × Condition	**14.35**	**4.61**	**61.02**	**3.11**	0.003**
Relative duration difference	Group	**10.12**	**4.90**	**58.94**	**2.07**	0.04*
	Condition	**42.86**	**4.67**	**60.96**	**9.18**	**<** 0.001***
	PPVT-R	**–0.63**	**0.22**	**58.88**	**–2.90**	0.005**
	Age	0.80	1.68	58.98	0.48	0.63
	Group × Condition	8.53	4.67	60.96	1.83	0.07
Time errors	Group	**0.15**	**0.07**	**68.00**	**2.08**	0.04*
	Condition	**–0.48**	**0.07**	**69.65**	**–6.68**	**<** 0.001***
	PPVT-R	–0.005	0.003	58.92	–1.67	0.10
	Age	–0.001	0.02	59.04	–0.44	0.67
	Group × Condition	**0.14**	**0.06**	**61.13**	**2.15**	0.04*

Bold values indicate statistical significance at *p* < 0.05. *p < 0.05, **p < 0.01, ***p < 0.001.

### Relative duration difference

Figure 4(b) shows the distribution of the relative duration differences for each group in both the Speech and Music conditions. The linear mixed-effects model revealed significant main effects of Group (*B* = 10.12, SE*B* = 4.90, *t*(58.94) = 2.07, *p* = 0.04) and Condition (*B* = 42.86, SE*B* = 4.67, *t*(60.96) = 9.18, *p* < 0.001). Both groups showed larger relative duration differences in the music condition than in the speech condition, and the autism group produced larger relative duration differences than did the non-autism group not only in the music condition (Autism: M(*SD*) = 163.15(129.26); Non-Autism: M(*SD*) = 116.66(80.55)) but also in the speech condition (Autism: M(*SD*) = 60.64(53.99); Non-Autism: M(*SD*) = 48.2(30.89)). Similarly, receptive vocabulary was a significant predictor of performance on relative duration matching (*B* = −0.63, SE*B* = 0.22, *t*(58.88) = −2.90, *p* = 0.005): the larger the receptive vocabulary of the participants, the greater the accuracy in their relative duration matching. The interaction between Group and Condition, and the effect of Age were not significant (see Table 4). Pearson correlations confirmed the significant association between the relative duration differences and PPVT-*R* (*r*(124) = -0.26, *p* = 0.003), but not with age (*r*(124) = 0.007, *p* = 0.94).

### Number of time errors

Figure 4(c) shows the distribution of the number of time errors for each group in both the Speech and Music conditions. The linear mixed-effects model revealed significant main effects of Group (*B* = 0.15, SE*B* = 0.07, *t*(68) = 2.08, *p* = 0.04) and Condition (*B* = -0.48, SE*B* = 0.07, *t*(69.65) = -6.68, *p* < 0.001). The interaction between Group and Condition was also significant (*B* = 0.14, SE*B* = 0.06, *t*(61.13) = 2.15, *p* = 0.04). Post hoc analyses with Holm-Bonferroni correction for multiple comparisons suggested that both groups showed fewer time errors in music imitation than in speech imitation (Autism: *t*(70.6) = 3.61, *p* < 0.001; Non-Autism: *t*(69.8) = 6.28, *p* < 0.001), and the autism group performed worse than the non-autism group in the music condition (*t*(128) = -2.98, *p* = 0.003, Autism: M(*SD*) = 33.67(16.53); Non-Autism: M(*SD*) = 20.7(15.31)), but not in the speech condition (*t*(129) = -0.12, *p* = 0.90, Autism: M(*SD*) = 46.27(11.22); Non-Autism: M(*SD*) = 45.03(10.63)). The effects of PPVT-R and Age did not reach significance (see Table 4).

## Discussion

Using matched speech and song stimuli, the present study investigated vocal imitation in Mandarin-speaking autistic and non-autistic individuals. Our acoustic analysis unveiled distinct patterns in vocal imitation performance between the two groups.

For speech imitation, Mandarin-speaking autistic participants were less accurate than non-autistic individuals in matching relative pitch and duration. For song imitation, they showed reduced performance on both relative and absolute duration matching. These results are inconsistent with the patterns observed in English speakers (Wang, Pfordresher, et al., 2021), where English-speaking autistic individuals exhibited differences with absolute but not relative pitch and duration matching in both speech and music conditions. Specifically, we did not observe reduced absolute pitch matching in Mandarin-speaking autistic individuals, for either speech or song, contrary to the evidence presented by English-speaking individuals. The reason for this may be related to the tone language background. Indeed, Deutsch et al. (2004) found that tone language speakers display a remarkably precise and stable form of absolute pitch when reproducing words. This may be because absolute pitch originally evolved as a feature of speech, similar to other features such as vowel quality, and speakers of tone languages naturally acquire this feature during critical periods of speech acquisition (Deutsch et al., 2004). Moreover, when using machine learning-based analysis to differentiate speech produced by autistic and non-autistic individuals, variations of voice pitch (e.g. absolute features) were significant between the two groups only for English speakers but not for Cantonese speakers (Lau et al., 2022). Thus, our Mandarin-speaking autistic participants, despite their relatively smaller receptive vocabularies compared to their peers, still had the advantage of a tone language background and showed comparable performance to non-autistic participants in terms of absolute pitch matching.

Regarding duration matching, the present findings complement those of Lau et al. (2022), where both English- and Cantonese-speaking autistic individuals exhibited atypical rhythm production relative to non-autistic individuals. Our results from Mandarin speakers further reveal that such rhythmic differences may be primarily driven by relative rather than absolute duration-matching abilities. In contrast, for English speakers, speech rhythm differences between autistic and non-autistic groups were evident in absolute rather than relative duration matching (Wang, Pfordresher, et al., 2021). Consequently, although differences with speech duration matching are shared across linguistic groups in autism, the underlying cause as related to absolute versus relative duration feature matching may vary across languages. In addition, consistent with previous studies (Carello et al., 2002; Ladányi et al., 2020), the current results showed that participants with higher receptive vocabulary abilities performed better in imitating the absolute and relative duration of notes/syllables. This relationship suggests that a larger receptive vocabulary may be linked to better temporal processing and timing control, which are crucial for accurate duration imitation and speech production. Therefore, future research should incorporate receptive verbal skills, along with expressive language, to provide a more holistic understanding of language abilities and their impact on duration imitation skills. Consistent with the hypothesis linking linguistic and musical rhythm (Patel & Daniele, 2003; Patel et al., 2006), atypical duration matching in the autism group was observed not only in speech but also in song imitation.

In terms of the research questions posed and our predictions, our finding of reduced duration matching but intact pitch matching during song imitation in autism is consistent with our hypothesis. Contrary to our hypothesis, however, both reduced relative pitch and duration matching were present during speech imitation in autism. This finding is to some extent in line with previous results showing atypical pitch and duration production of speech in autism (Chen et al., 2022; Fosnot & Jun, 1999; Hubbard & Trauner, 2007). Our results further indicate that imitation differences in speech might only be observed in relative rather than absolute features in Mandarin-speaking autistic individuals. As speaking a tone language is one of the most robust ways to improve the ability to process pitch, including both perception and production (Bidelman et al., 2013; Burnham et al., 2015; Creel et al., 2018; Li et al., 2021; Pfordresher & Brown, 2009), we hypothesized that experience with a native tone language might have a compensatory effect on possible pitch matching difficulties in Mandarin-speaking autistic individuals. That is, we expected that in the current imitation tasks, autistic participants would show reduced duration but not pitch imitation in both speech and song compared to non-autistic participants. However, the results revealed that this compensatory effect appears to be present only when imitating song stimuli.

To the best of our knowledge, pitch and duration matching in speech and song imitation has not been previously studied in Mandarin-speaking autistic individuals, making it difficult to find evidence to explain why Mandarin-speaking autistic individuals show preservation of relative pitch in music but not in a speech during vocal imitation. One possibility might relate to the different precision requirements for pitch processing between speech and music. There has been ample evidence suggesting that, to achieve adequate communication, a higher degree of pitch precision is required in conveying musical meaning than speech meaning (F. Liu et al., 2013; Patel, 2008, 2011). Indeed, the present study, together with previous studies (F. Liu et al., 2013; Mantell & Pfordresher, 2013; Wang, Pfordresher, et al., 2021), found that both autistic and non-autistic individuals imitated song more accurately than speech on all pitch-related measures. Thus, the compensatory effect of experience with a native tone language on autistic individuals seems to work only when pitch precision is required, as in music; but not when pitch approximation is needed, as in speech. The inactivated compensatory effect of pitch in speech led to reduced performance in the autism group compared to the non-autism group. Another possibility may be linked to the multi-role of pitch in tone languages. As aforementioned, unlike in intonation languages, the imitation of pitch in tone languages occurs in parallel including prosodic meaning at the sentence level and lexical meaning at the syllable or word level, which increases the complexity and difficulty of pitch imitation in the speech condition (F. Liu & Xu, 2005; Yuan, 2011). Finally, extensive research has shown a dissociation between musical (enhanced or intact) and linguistic (reduced) skills in autism (for reviews, see O’Connor, 2012; Ouimet et al., 2012; Quintin, 2019). Autistic individuals also show typical brain activations and connectivity to musical stimuli but not to speech stimuli (Lai et al., 2012; Sharda et al., 2015). Thus, typical pitch imitation for songs among autistic Mandarin speakers is in line with the existing wider literature. Further studies are needed to explore these possibilities.

Interestingly, autistic participants made more pitch contour errors than non-autistic participants across speech and music domains. There are four lexical tones in Mandarin, high level, high rising, falling-rising, and high falling, which correspond to four different shapes of pitch contour (Howie, 1976). Research has found that Mandarin speakers are more sensitive to pitch contours than speakers of intonation languages (Huang & Johnson, 2011; Li et al., 2021; Xu et al., 2006). In addition, a recent study examined the pitch production of Cantonese tones (CT) in Cantonese- and Mandarin-speaking autistic and non-autistic children (Chen et al., 2022). They found that autistic children exhibited atypical pitch production for contour tones with steeper slopes (i.e. CT25 in the study) but not for level tones (i.e. CT55, CT33, and CT22) or contour tones with flatter slopes (i.e. CT21, CT23). In the present study, pitch contours were defined based on the pitch heights of two consecutive syllables/notes: up or down if the difference in pitch interval was higher or lower by 50 cents or more; otherwise, flat. Each participant had 60 values of pitch contour errors for each condition. Out of a total of 120 values created by the male/female model, only six were flat contours. Thus, the current results extended the findings of Chen et al. (2022), suggesting that autistic children who speak a tone language might differ in producing pitch contours across syllables in both speech and music domains compared to their peers. In addition, older participants were associated with fewer pitch interval errors, suggesting that age-related maturation positively influences the accuracy of pitch interval imitation. Evidence from the music domain suggests that there are learning and transfer effects in vocal matching of pitch intervals (Harvey et al., 1987), which aligns with our findings. However, the effect of age was only observed in the matching of pitch intervals among the pitch-related parameters, indicating that these results should be interpreted with caution and warrant further investigation.

Moreover, in line with previous studies (F. Liu et al., 2013; Mantell & Pfordresher, 2013; Patel, 2008, 2011) both autistic and non-autistic Mandarin speakers showed greater sensitivity to duration in speech than in song, while exhibiting greater sensitivity to pitch in song compared to speech. This suggests that pitch imitation is independent of the imitation of duration across different domains (speech vs music) (Dalla Bella et al., 2007, 2009; Drake & Palmer, 2000; Mantell & Pfordresher, 2013). These results support previous findings in perception research that suggest the perception of speech content is most affected by degradation in the temporal dimension, while the perception of melodic content is most affected by degradation in the spectral dimension (Albouy et al., 2020).

While our study provides valuable insights into vocal imitation in autistic individuals within tonal linguistic contexts, several limitations should be acknowledged. First, due to our task demands, we recruited participants whose cognitive functioning lay on the typical to the higher end of the distribution on the autism spectrum. This limited the generalizability of our current findings to individuals with cognitive disadvantages, a research area that remains to be explored. In addition, given the severe shortage of reliable and standardized speech and language assessment tools available in the Chinese language, especially in Mandarin (Jin & Zhu, 2023), the PPVT-R was chosen to measure receptive vocabulary skills. While the PPVT-R is a well-established instrument for assessing vocabulary, it focuses specifically on receptive vocabulary and does not fully capture the participants’ overall language abilities. In particular, without a measure of expressive language, we cannot rule out the possibility that group differences may be influenced by variations in expressive language abilities. It should also be noted that due to the limitation of available Chinese norms of the PPVT-R, we supplemented our analysis with the American norms (Dunn & Dunn, 1981) for standardization purposes. This reliance on over 40-year-old norms may explain the higher receptive vocabulary abilities observed in our sample. Future research would benefit from the development and validation of comprehensive assessments of both receptive and expressive language, as well as pragmatic skills that are tailored to the linguistic characteristics of the Mandarin-speaking population (Zhang et al., 2021) to provide a more holistic understanding of language abilities and vocal imitation skills. Finally, the age range of our participants was relatively wide, including both children and adolescents. While we incorporated age as a factor in the statistical analysis to account for potential age-related variations, the observed nonsignificant age effects in most results suggest that, within the current sample, age may not be a prominent factor influencing vocal imitation abilities. However, it is crucial to recognize that puberty introduces substantial alterations to the vocal apparatus, along with developmental changes in the vocal tract and vocal fold length (Harries et al., 1997). Despite our efforts to control for age-related differences, the variability in the timing and the extent to which development-related voice changes may contribute to the nuanced outcomes in vocal imitation remains to be assessed. Future investigations with a more refined age focus or additional measures to directly assess and control for development could offer a more comprehensive understanding of the intricate interplay between vocal imitation abilities in autism and developmental changes.

Finally, it is worth exploring the potential clinical relevance of the current results on relative versus absolute feature matching during vocal imitation in autism. Research has shown that effective imitation of vocal features enhances language acquisition in both typical development (Kuhl & Meltzoff, 1996; Masur & Olson, 2008) and in autism (Ross & Greer, 2003; Tarbox et al., 2009). It has been suggested that social reinforcement through caregivers’ vocal imitation can facilitate infants’ vocalizations (Neimy et al., 2017; Pelaez et al., 2018), and slowing down the presentation of vocal sounds can better induce vocal imitation in autistic children (Tardif et al., 2007). Thus, autistic children’s language learning may benefit from vocal imitation of sung materials, an area of research that warrants experimental investigations.

## Conclusion

This study assessed, for the first time, the vocal imitation ability of Mandarin-speaking autistic individuals, using speech and song stimuli matched for linguistic content and pitch contour. The results indicated that Mandarin-speaking autistic individuals showed atypical duration but not pitch matching during song imitation, whereas for speech imitation only relative but not absolute pitch and duration matching was atypical. In addition, Mandarin-speaking autistic individuals showed differences in imitating pitch contours across speech and song. These findings reveal a vocal imitation atypicality across speech and music domains among Mandarin-speaking autistic individuals, with a unique pattern that differs from previous studies focusing on non-tonal language speakers. This study therefore extends our understanding of vocal imitation in autism across different languages. Future research should examine vocal imitation from other linguistic contexts to consolidate the current results.

## Supplemental Material

sj-docx-1-aut-10.1177_13623613241275395 – Supplemental material for Atypical vocal imitation of speech and song in autism spectrum disorder: Evidence from Mandarin speakers Supplemental material, sj-docx-1-aut-10.1177_13623613241275395 for Atypical vocal imitation of speech and song in autism spectrum disorder: Evidence from Mandarin speakers by Li Wang, Peter Q Pfordresher, Cunmei Jiang and Fang Liu in Autism
