# Supplementary material for: Atypical vocal imitation of speech and song in autism spectrum disorder: Evidence from Mandarin speakers
Source: Autism. 2024 Sep 6;29(2):408–23. doi: 10.1177/13623613241275395 (PMC11816480; doi:10.1177/13623613241275395)
Supplement: sj-docx-1-aut-10.1177_13623613241275395 – Supplemental material for Atypical vocal imitation of speech and song in autism spectrum disorder: Evidence from Mandarin speakers [file sj-docx-1-aut-10.1177_13623613241275395.docx]

**Atypical vocal imitation of speech and song in autism spectrum disorder: Evidence from Mandarin speakers**

**Supplementary material**

**Supplementary Table 1**. Musical notations of the sung stimuli used in the experiment.

| Musical stimuli corresponding to speech stimuli with an early focus | Musical stimuli corresponding to speech stimuli with a late focus |
| --- | --- |
| 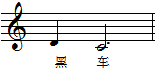 | 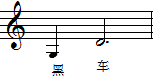 |
| 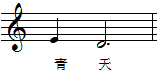 | 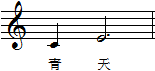 |
| 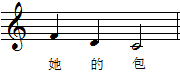 | 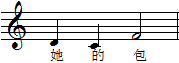 |
| 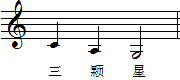 | 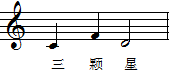 |
| 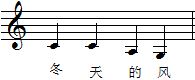 | 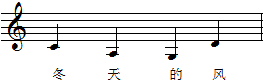 |
| 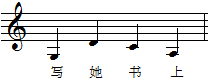 | 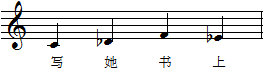 |
| 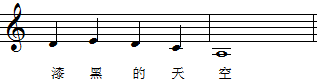 | 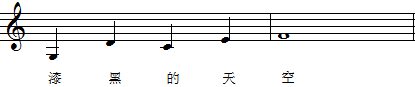 |
| 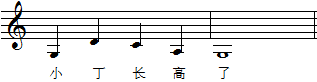 | 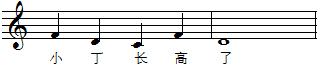 |
| 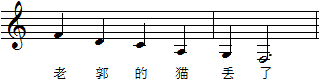 | 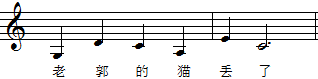 |
| 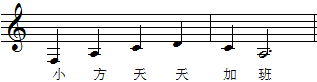 | 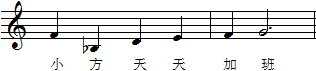 |

**Supplementary Table 2**. Coefficients for linear mixed-effects models for Gender effects across different imitation measures.

| **Measure** | **Effect** | **Estimate** | **Std. Error** | **df** | ***t*** | ***p*** |
| --- | --- | --- | --- | --- | --- | --- |
| Absolute pitch deviation | Gender | 4.13 | 24.83 | 61.03 | 0.17 | 0.87 |
| Relative pitch deviation | Gender | -6.35 | 13.94 | 60.58 | -0.46 | 0.65 |
| Pitch contour errors | Gender | 0.05 | 0.06 | 59.73 | 0.76 | 0.45 |
| Pitch interval errors | Gender | 0.01 | 0.09 | 60.96 | 0.16 | 0.87 |
| Absolute duration difference | Gender | -2.35 | 15.23 | 60.29 | -0.15 | 0.88 |
| Relative duration difference | Gender | 4.73 | 14.51 | 60.62 | 0.33 | 0.75 |
| Time errors | Gender | -0.10 | 0.18 | 60.84 | -0.57 | 0.57 |
